# A method for filamentous fungal growth and sample preparation aimed at more consistent MALDI-TOF MS spectra despite variations in growth rates and/or incubation times

**DOI:** 10.1093/biomethods/bpz003

**Published:** 2019-05-03

**Authors:** Michael A Reeve, Denise Bachmann

**Affiliations:** CABI, Bakeham Lane, Egham, Surrey, UK

**Keywords:** Acid-soluble proteins, fungal growth, liquid culture, paper-supported culture, spectral consistency

## Abstract

Matrix-assisted laser-desorption and ionization time-of-flight mass spectrometry can be used for the characterization and identification of filamentous fungi, for which it is desirable to have a means of growth in which the resulting spectra remain as consistent as possible over time. To this end, we initially opted for growth in oil-overlaid small-volume liquid culture, using a medium (Czapek Dox) not containing significant amount of proteins or peptides, and with protein extraction from the entire culture volume. For both 3-week and 10-day time courses, however, we observed marked spectral changes over growth time, along with lower peak richness compared to agar-plate controls. Guided by the above, we next employed a more nutrient-rich MALDI-TOF MS-compatible liquid-culture medium, now used without an oil overlay. For a 10-day time course, we again observed marked spectral changes over growth time, along with lower peak richness compared to agar-plate controls. Finally, we opted for a method employing filter-paper-supported growth in the same MALDI-TOF MS-compatible rich medium within sealed 1.5 ml Eppendorf tubes, again with protein extraction from the entire culture volume. Using this final method, while we observed significant spectral changes between 2 days and 3 days, from 3 days to 10 days the spectra remained very consistent, with comparable peak richness to agar-plate controls. This method gave slightly better identifications and lower spectral variance compared to agar-plate controls, and the use of this method for the construction of growth-time-point-specific databases for fungal identification is discussed.

## Introduction

Matrix-assisted laser-desorption and ionization time-of-flight mass spectrometry (MALDI-TOF MS) is a powerful tool for the characterization and/or identification of biological samples. In this versatile technique, the MALDI soft ionization process [1] enables the preparation of large proteins intact in the gas phase, with predominantly a single positive charge [2]. When such a charged protein is accelerated along a tube held at high vacuum by means of an electrical field, the time-of-flight is proportional to the square root of the mass-over-charge ratio for the protein [3] and, using this simple relationship, a mass spectrum can be generated for the protein components in a particular biological sample [3]. For the characterization and identification of samples, the mass spectrum of a subset of the expressed proteome is often used (normally the highly expressed acid-soluble proteins, believed to include many ribosomal proteins) [4]. For the MALDI-TOF MS analysis of filamentous fungi, it is desirable to have not only a means of protein extraction and MALDI-TOF MS sample preparation that is reliable, cheap, and simple to perform but also a means of fungal growth in which the resulting MALDI-TOF MS spectra remain as consistent as possible despite real-life variations in fungal growth rates and/or incubation times.

For protein extraction and MALDI-TOF MS sample preparation, human clinical microbiology, in particular the diagnosis of bacterial and yeast infections, has been a key driving force behind past protocol development [4]. This area is comprehensively reviewed by Clark *et al.* [3], along with much of the underlying theory and many of the methods commonly used for MALDI-TOF MS sample preparation. Additional methods have also been developed for mycoplasma [5], yeasts [6] and filamentous fungi [7–9]—particularly the ‘full-extraction’ protocols after the work of Cassagne *et al.* [10]. ‘Direct-transfer’ protocols (reviewed in [3]) are commonly used for the identification of bacteria and yeasts. In these methods, material from a single microbial colony from an agar plate is first smeared on a sample-plate target zone (normally using a sterile toothpick). The microbial biomass is then overlaid with MALDI matrix [frequently, α-cyano-4-hydroxycinnamic acid (HCCA)] in aqueous acetonitrile containing trifluoroacetic acid (TFA), followed by drying and loading into the mass spectrometer. In some protocols, an additional overlay of 70% (v/v) aqueous formic acid followed by drying is also carried out prior to the overlay of matrix. ‘Full-extraction’ protocols based on the method of Cassagne *et al.* [10, 11] are generally used for filamentous-fungal identifications. In these methods, fungal biomass is first placed in water and ethanol is added to around 70% (v/v) followed by centrifugation. The pellet is then incubated in 70% (v/v) aqueous formic acid to which an equal volume of acetonitrile is subsequently added, followed by further centrifugation. Supernatant is then pipetted onto the sample plate target zone and dried. This is overlaid with MALDI matrix, followed by drying and loading into the mass spectrometer. In terms of applicability, direct-transfer protocols are rapid to perform but are not well suited to the transfer of some types of fungal biomass, particularly powdery forms of mycelium and spores. In contrast, full-extraction protocols are easier in this regard but they are however vulnerable to loss of biomass after the first centrifugation step when small amounts of material are used. Due to their reliance upon centrifugation, full-extraction protocols can also be limited in terms of throughput by centrifuge availability and/or capacity. In response to the above, Reeve *et al.* have developed a highly simplified and inexpensive method for MALDI-TOF MS sample preparation with broad applicability to bacteria, fungi, insects and plants [12, 13] that lyses cells by immersion or maceration in aqueous acetonitrile containing TFA to selectively extract acid-soluble proteins, with lysis and extraction carried out in the presence of near-saturated and inexpensive-grade MALDI matrix. The resulting matrix-saturated lysate containing acid-solubilized proteins is then simply dried down directly onto the MALDI-TOF MS sample plate and analysed. In the current article, we have used this method for protein extraction and MALDI-TOF MS sample preparation from fungal samples.

For filamentous fungi, significant variation in MALDI-TOF MS spectra can be observed under different growth conditions on agar plates. Supplementary Figure S1 shows an illustrative example of this, in which spectra are shown that derive from the same strain (CAV 789 [14]) of *Fusarium oxysporum* f.sp *cubense* grown for 1 week at 25°C on agar plates containing two different media. The spectra in Supplementary Figure S1 demonstrate that the composition of the media has a significant influence on the protein expression, with the observed differences being perhaps too great to support credibly the hypothesis that fungal MALDI-TOF MS spectra are based largely on ‘housekeeping’ proteins derived predominantly from ribosomes. Variation in fungal MALDI-TOF MS spectra can also be observed over time for growth on agar plates containing the same medium. Supplementary Figure S2 shows an illustrative example of this, in which spectra are shown that derive from the same strain (CAV 293 [15]) of *Fusarium oxysporum* f.sp *cubense* grown at 25°C on agar plates containing SNA medium for 3 days, 6 days, 11 days, 16 days and 19 days. Temporal influences can therefore also have a significant effect on the MALDI-TOF spectrum, particularly the relative heights of the observed protein peaks.

The differentiation of fungi on agar plates and the adaptation to different growth media has, in the past, been used to aid their characterization [16] but this property adds additional and undesirable variance to MALDI-TOF MS-based analysis. It would therefore be of considerable benefit to be able to grow fungi in a manner that limited their adaptation and differentiation as much as possible. To this end, we initially investigated growth in liquid culture as an alternative to expanding surface growth on agar plates. Growth of fungi in liquid culture has already been used prior to MALDI-TOF MS sample preparation and indeed protocols are given in the Bruker manual [11] for large-volume liquid-culture growth. Such growth is not however without its difficulties because large-volume growth in liquid culture tends to give rise to highly viscous and localized clumps of cells within the culture medium, which can be very difficult to sample. In order to circumvent this problem, in ref. [11], large-volume liquid cultures are allowed to sediment, fungal biomass is then transferred to a fresh tube, and is subsequently recovered free of culture-medium protein and peptide components by centrifugation (which does not always result in a well-defined or compact pellet) before processing by full-extraction protocols [10, 11].

Having developed a protein-extraction and sample-preparation method that is not constrained by a requirement for centrifugation [12, 13], we sought to integrate this with a similar centrifugation-independent method for liquid-culture growth, with the aspiration of developing a method for MALDI-TOF MS analysis of fungi for which the resulting MALDI-TOF MS spectra remain as consistent as possible despite real-life variations in fungal growth rates and/or incubation times. To this end, our initial rationale was to grow fungi in very small volumes of liquid culture within capped Eppendorf tubes, with subsequent use of the *entire* liquid volume for MALDI-TOF MS sample preparation. In order to develop a centrifugation-independent and integrated method for small-volume liquid-culture growth followed by protein extraction and MALDI-TOF MS sample preparation by adding a single reagent comprising acetonitrile, TFA and HCCA matrix, the culture medium used for liquid-phase growth should not contain proteins or peptides that will contaminate the resulting MALDI-TOF MS spectra. Numerous protein-free and peptide-free fungal-culture media have been described [17]. Some of these are genera-specific (e.g. SNA medium for *Fusarium* spp. [18]) but some are also suitable for the growth of a wide range of filamentous fungi [19]. Czapek Dox medium [20] is one example of the latter, and was therefore selected for use in the current study. In this semisynthetic medium, sucrose is the sole source of carbon and sodium nitrate is the sole source of nitrogen, dipotassium phosphate acts as a buffer, and magnesium sulphate, potassium chloride and ferrous sulphate provide essential ions for fungal growth. For further growth-medium development, nutrient supplements (e.g. amino-acid sources) can be assessed empirically for their suitability to methods of the type described above by MALDI-TOF MS analysis of non-inoculated samples of test growth media. Given the likelihood of extended incubation times in very small liquid droplets, bacterial overgrowth of fungal cultures (which would add unwanted peaks to the observed MALDI-TOF MS spectra) might be difficult to detect within capped Eppendorf tubes and so we opted to negate this possibility through the inclusion of antibiotics (chloramphenicol and penicillin) in the culture media used throughout these studies. Finally, in order to test our initial method and all subsequent iterations, we employed a representative strain of a plant-pathogenic fungus that was readily available to us from the CABI Genetic Resource Collection [21].

## Materials and methods

### Mass spectrometry

Mass spectrometry covering the range 2–20 kDa was carried out using a Bruker Microflex LT linear-mode instrument running the MALDI Biotyper 4.0 applications (Bruker Daltonik, Bremen, Germany), using a 60 Hz frequency and 3 ns pulse-duration nitrogen laser (70 µJ, with maximum output 225 µJ), with a wavelength of 337 nm and spot size of 100 µm, with 240 laser shots per sample. The laser settings were Global Attenuator Offset (0%), Attenuator Offset (25%) and Attenuator Range (30%), and the ion-source voltage was 19.98 kV. Bruker MBT Biotarget 96 plates (Bruker ref. 1840375) were used for all samples in this study. Calibration was carried out using the manufacturer’s ‘BTS’ controls (*Escherichia coli* proteins supplemented with ribonuclease A and myoglobin), using peaks with masses at 3, 637.8; 5, 096.8; 5, 381.4; 6, 255.4; 7, 274.5; 10, 300.2; 13, 683.2, and 16, 952.3 for calibration according to the manufacturer’s instructions. Spectra were acquired using MALDI Biotyper RTC Version 4.0 (Build 19) using the manufacturer’s standard settings (Centroid peak-detection algorithm and TopHat baseline subtraction). Database entries were made as single-spectra MSPs using the Bruker Online Client software suite (Version 4.0.19, Bruker Daltonik, Bremen, Germany), again using the manufacturer’s standard settings. For spectral comparisons, Bruker identification scores were derived using the standard Bruker algorithm. This first converts raw mass spectra into peak lists, which are then compared between spectra. Three separate values are computed: the number of peaks in the reference spectrum that have a closely matching partner in the test spectrum (value range 0–1), the number of peaks in the test spectrum that have a closely matching partner in the reference spectrum (value range 0–1), and the peak-height symmetry of the matching peaks (value range 0–1). The above three values are multiplied together and normalized to 1000, and the base-10 logarithm is then taken to give the final Bruker score (range 0–3). Bruker scores of scores between 2.3 and 3.0 indicate very close relatedness, scores between 2.0 and 2.3 indicate close relatedness, and scores below 1.7 indicate low relatedness. All spectra are shown baseline-subtracted, smoothed, *y*-axis-autoscaled and covering the mass range 2 kDa to 20 kDa (with *x*-axis scale increments of 2 kDa).

### Fungal strains

*Penicillium digitatum* (IMI 380881, from host *Citrus sinensis*) was used in this study.

### Culture conditions

Czapek Dox medium was made up as 30 g/l sucrose, 2 g/l sodium nitrate, 1 g/l dipotassium phosphate, 0.5 g/l magnesium sulphate, 0.5 g/l potassium chloride, 0.01 g/l ferrous sulphate, pH 7.3. Czapek Dox (CZ) agar was made up as 30 g/l sucrose, 2 g/l sodium nitrate, 1 g/l dipotassium phosphate, 0.5 g/l magnesium sulphate, 0.5 g/l potassium chloride, 0.01 g/l ferrous sulphate, 15 g/l agar, pH 7.3. Potato dextrose agar (PDA) (Oxoid) was made up as 4 g/l potato extract, 20 g/l glucose, 15 g/l agar, pH 5.6. ‘Supplements-and-dextrose medium’ (SDM) was made up as 30 g/l sucrose, 10 g/l glucose, 5 g/l bacterial peptone, 5 g/l yeast extract, 2 g/l sodium nitrate, 1 g/l dipotassium phosphate, 0.5 g/l magnesium sulphate, 0.5 g/l potassium chloride, 0.01 g/l ferrous sulphate, pH 7.3. Penicillin G and chloramphenicol were both used at 75 µg/ml final concentration in broth and agar.

### Reagents and paper

≥99.8% ethanol, ≥ 98% (TLC-grade) α-cyano-4-hydroxycinnamic acid (HCCA) matrix, LC-MS-grade acetonitrile, 99% ReagentPlus^®^-grade TFA, molecular-biology grade light mineral oil, and Whatman^®^ qualitative filter paper, Grade 3 (90 mm circles), were purchased from Sigma (Gillingham, UK). CHROMASOLV^TM^ LC-MS-grade water was purchased from Fluka (Loughborough, UK).

## Protein extraction and MALDI-TOF MS sample preparation

### Time course #1

For fungal cultures grown on agar plates, fungal biomass was mixed with 70 µl of (11 mg/ml HCCA matrix in 65% (v/v) acetonitrile, 2.5% (v/v) TFA, and 32.5% (v/v) water) using a plastic inoculating loop. One microlitre of the resulting crude lysate was then pipetted onto the Bruker sample plate, air dried, and loaded into the spectrometer. For fungal cultures grown in small-volume liquid culture, fungal biomass was grown in 5 µl spore-suspension-inoculated liquid aliquots beneath an overlay of 20 µl of light mineral oil in capped 1.5 ml Eppendorf tubes. 65 µl of (11.8 mg/ml HCCA matrix in 70% (v/v) acetonitrile, 2.7% (v/v) TFA, and 27.3% (v/v) water) were then mixed with the culture medium and 1 µl of the resulting crude lysate was pipetted onto the Bruker sample plate, air dried, and loaded into the spectrometer. Samples inoculated at *t* = 0 were analysed by MALDI-TOF MS to ensure that the biomass used for inoculum (154 spores/µl in the starting culture) was insufficient to generate spectra without further growth. Negative controls were also carried out as described above but using non-inoculated medium. Duplicate sample preparations were carried out for each strain and culture-condition combination at 0 weeks, 1 week, 2 weeks and 3 weeks incubation at 23°C. Replicate one sample preparations for each strain and culture-condition combination were used to construct database mass spectra (as single-spectra MSPs using the Bruker Online Client software). The replicate 2 ‘test’ samples were then compared to the replicate one ‘reference’ database spectra, again using the Bruker Online Client software suite as described above.

### Time course #2

For fungal cultures grown on agar plates, fungal biomass was mixed with 50 µl of 11 mg/ml HCCA matrix in 65% (v/v) acetonitrile, 2.5% (v/v) TFA and 32.5% (v/v) water using a plastic inoculating loop. One microlitre of the resulting crude lysate was then pipetted onto the Bruker sample plate, air dried and loaded into the spectrometer. For fungal cultures grown in small-volume liquid culture, fungal biomass was grown in 10 µl spore-suspension-inoculated liquid aliquots beneath an overlay of 30 µl of light mineral oil in capped 1.5 ml Eppendorf tubes. 40 µl of [13.8 mg/ml HCCA matrix, 81.3% (v/v) acetonitrile], 3.13% (v/v) TFA and 15.6% (v/v) water were then mixed with the culture medium and 1 µl of the resulting crude lysate was pipetted onto the Bruker sample plate, air dried and loaded into the spectrometer. Samples inoculated at *t* = 0 were again analysed by MALDI-TOF MS to ensure that the biomass used for inoculum (26 spores/µl in the starting culture) was insufficient to generate spectra without further growth. Negative controls were also carried out as described above but using non-inoculated medium. Duplicate sample preparations were carried out for each strain and culture-condition combination at 1 day, 2 days, 3 days, 8 days and 10 days incubation at 23°C. Replicate 1 sample preparations for each strain and culture-condition combination were used to construct database mass spectra (as single-spectra MSPs using the Bruker Online Client software). The replicate 2 ‘test’ samples were then compared to the replicate 1 ‘reference’ database spectra, again using the Bruker Online Client software suite.

### Time course #3

Samples were treated as for time course #2 except that the oil overlay was omitted and duplicate sample preparations were carried out for each strain and culture-condition combination at 2 days, 3 days, 7 days and 10 days incubation at 23°C.

### Time course #4

Samples were treated as for time course #3 except the 10 µl liquid aliquots were absorbed into 6 mm diameter discs of Whatman^®^ qualitative filter paper, Grade 3 (prepared using a hole punch) prior to incubation in capped 1.5 ml Eppendorf tubes.

## Results

### Time course #1

**Figure 1:**
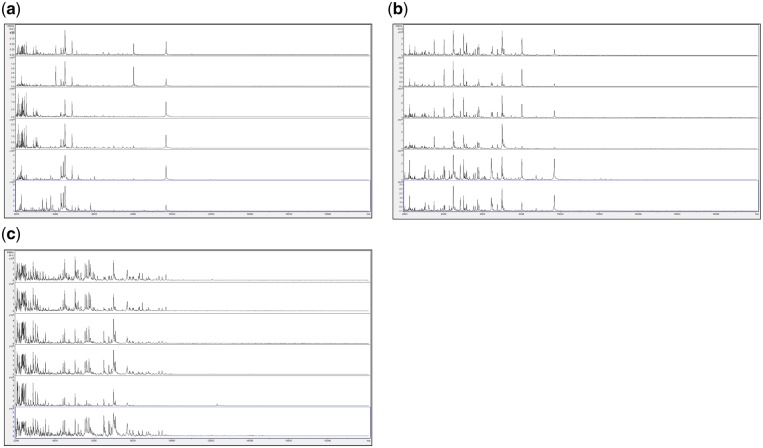
MALDI-TOF MS spectra obtained from *P. digitatum* IMI 380881 grown for 1 week, 2 weeks and 3 weeks at 23°C (a) in 5 µl droplets of CZ medium overlaid with 20 µl of light mineral oil in 1.5 ml Eppendorf tubes, (b) on CZ agar plates and (c) on PDA plates. Each panel shows samples, from top to bottom, after 1 week (replicates 1 and 2), after 2 weeks (replicates 1 and 2) and after 3 weeks (replicates 1 and 2).

We initially reasoned that growth of fungi on agar plates encourages adaptation and differentiation that adds significantly to MALDI-TOF MS spectral variance and, instead, initially opted for growth in liquid culture. In order to circumvent problems caused by regional clumping frequently observed in large-volume liquid culture, we opted for growth in very small volumes and then protein extraction from the *entire* culture volume. Having developed a protein-extraction and sample-preparation method that is not constrained by a requirement for centrifugation, we sought to integrate this with a similar centrifugation-independent method for liquid-culture growth, and so we opted for a protocol in which the above combined protein-extraction and MALDI-TOF MS sample preparation reagent could be added directly to the small culture volume, therefore necessitating the use of a culture medium such as CZ not containing significant amounts of proteins or peptides (which would then contaminate the MALDI-TOF MS spectra). In order to negate bacterial overgrowth of fungal cultures (which would also add unwanted peaks to the MALDI-TOF MS spectra), we also included antibiotics in the culture media used throughout these studies. Following the above rationale, we first grew fungi for up to 3 weeks in very small volumes of CZ medium containing antibiotics, under an oil overlay within capped Eppendorf tubes, with subsequent use of the entire liquid volume for MALDI-TOF MS sample preparation. Figure 1 shows the MALDI-TOF MS spectra obtained from *Penicillium digitatum* IMI 380881 grown for 1 week, 2 weeks and 3 weeks at 23°C in 5 µl droplets of CZ medium overlaid with 20 µl of light mineral oil in 1.5 ml Eppendorf tubes, on CZ agar plates, and on potato dextrose agar (PDA) plates.

As can be seen in Figure 1, for the samples grown in 5 µl droplets of CZ medium overlaid with 20 µl of light mineral oil in 1.5 ml Eppendorf tubes (Fig. 1(a)), the spectra change markedly over the time course, with peaks of around 8000 Da and 4000 Da that are only visible at 1 week and a cluster of peaks with mass between 2000 Da and 3000 Da that are more abundant at 2 weeks. For the samples grown on CZ agar plates (Fig. 1(b)), the spectra again change over the time course, with peaks of around 6400 Da and 9800 Da that are more abundant at 3 weeks. For the samples grown on PDA plates (Fig. 1(c)), the spectral consistency over the time course is better than for the samples grown in 5 µl droplets of CZ medium overlaid with 20 µl of light mineral oil in 1.5 ml Eppendorf tubes and the samples grown on CZ agar plates, with the only outlier being replicate 1 for the 3-week PDA plate sample, which shows a slightly weaker spectrum. The number of peaks observed for the samples grown on PDA plates is greater than for the samples grown on CZ plates, which is in turn greater than for the samples grown in 5 µl droplets of CZ medium overlaid with 20 µl of light mineral oil in 1.5 ml Eppendorf tubes. Duplicate negative controls were carried out for all time points and all culture conditions, and gave no spectra (data not shown). In addition, no sporulation was visible in any of the liquid cultures at any incubation time, despite vigorous sporulation of *P. digitatum* IMI 380881 on agar plates, even after very short incubation times.

**Figure 2:**
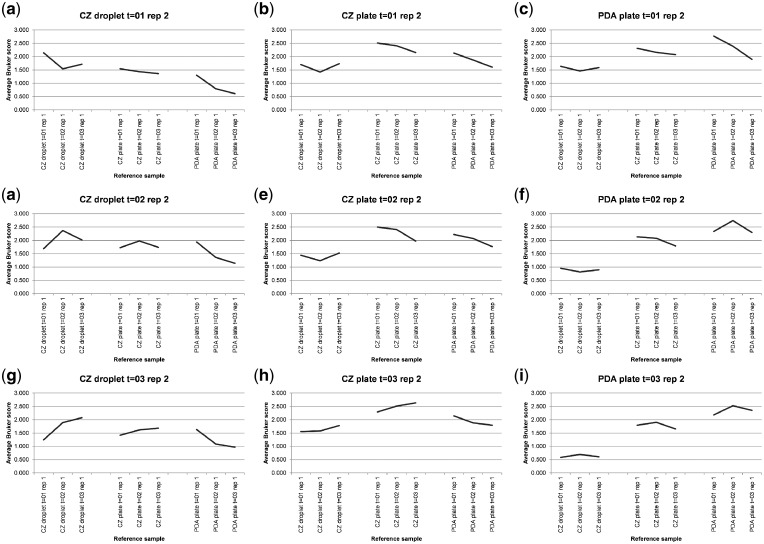
Bruker scores from comparisons between *P. digitatum* IMI 380881 replicate 1 ‘reference’ database spectra (*x*-axis labels) and replicate 2 ‘test’ sample spectra (graph titles).

Supplementary Table S1 shows MALDI-TOF MS spectral-comparison scores from the spectra shown in Fig. 1 between replicate 1 ‘reference’ database spectra and replicate 2 ‘test’ sample spectra for *P. digitatum* IMI 380881 and Fig. 2 shows Bruker scores using the data from Supplementary Table S1 for comparisons between replicate 1 ‘reference’ database spectra and replicate 2 ‘test’ sample spectra for *P. digitatum* IMI 380881.

As can be seen in Fig. 2, in seven out of nine cases (Fig. 2a–d and Fig. 2f–h), the highest Bruker score is observed for the same growth medium, growth conditions and growth time. In Fig. 2e, the highest Bruker score was obtained for the 1-week reference sample rather than the 2-week reference sample and in Fig. 2i, the highest Bruker score was obtained for the 2-week reference sample rather than the 3-week reference sample. For CZ agar-plate and CZ liquid-droplet test-sample spectral comparisons against PDA plate reference samples, in every case, the highest Bruker score is to the PDA plate grown for the shortest amount of time, suggesting that spectral divergence from CZ medium for samples grown of PDA plates increases with growth time. For CZ liquid-droplet test-sample spectral comparisons against CZ-plate reference samples, in every case, the highest Bruker score is to the CZ plate grown for the same length of time, suggesting that spectral divergence in CZ medium is similar over time in both liquid culture and on agar plates.

When used for comparison between an unknown test-sample spectrum and a database of known reference-sample spectra, the guideline interpretations for the Bruker scores used for ‘identification’ in this manner are: 2.300–3.000, ‘highly-probable species-level identification’; 2.000–2.299, ‘secure genus-level identification and probable species-level identification’; 1.700–1.999, ‘probable genus-level identification’; and 0.000–1.699, ‘no reliable identification’. Looking at the 3-week time course #1 data in this context, for the PDA-plates dataset, the highest Bruker score is 2.767 for the 1-week test spectrum against the 1-week reference spectrum and the lowest Bruker score is 1.896 for the 1-week test spectrum against the 3-week reference spectrum (a difference of 0.871 Bruker units, from ‘highly-probable species-level identification’ to ‘probable genus-level identification’). For the CZ-plate dataset, the highest Bruker score is 2.632 for the 3-week test spectrum against the 3-week reference spectrum and the lowest Bruker score is 1.976 for the 2-week test spectrum against the 3-week reference spectrum (a difference of 0.656 Bruker units, from ‘highly-probable species-level identification’ to ‘probable genus-level identification’). For the CZ-droplet dataset, the highest Bruker score is 2.371 for the 2-week test spectrum against the 2-week reference spectrum and the lowest Bruker score is 1.245 for the 3-week test spectrum against the 1-week reference spectrum (a difference of 1.126 Bruker units, from ‘highly-probable species-level identification’ to ‘no reliable identification’). Finally, for the entire dataset, the highest Bruker score is 2.767 for the 1-week PDA-plate test spectrum against the 1-week PDA-plate reference spectrum and the lowest Bruker score is 0.586 for the 3-week PDA-plate test spectrum against the 1-week CZ-droplet reference spectrum (a difference of 2.181 Bruker units, from ‘highly-probable species-level identification’ to ‘no reliable identification’).

### Time course #2

**Figure 3:**
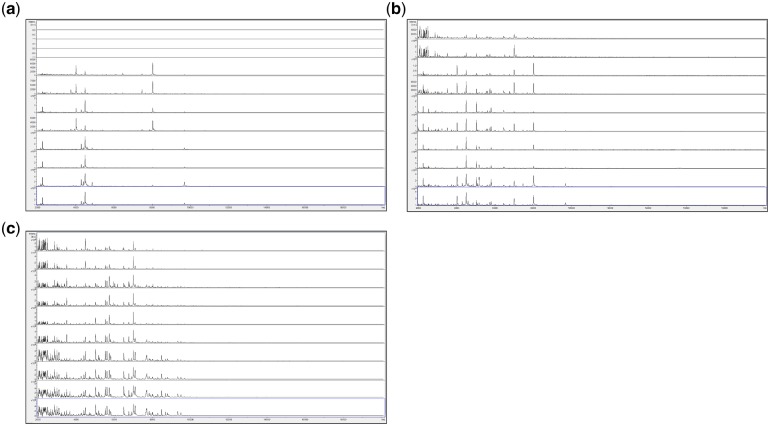
MALDI-TOF MS spectra obtained from *P. digitatum* IMI 380881 grown for 1 day, 2 days, 3 days, 8 days and 10 days incubation at 23°C (a) in 10 µl droplets of CZ medium overlaid with 30 µl of light mineral oil in 1.5 ml Eppendorf tubes, (b) on CZ agar plates and (c) on PDA plates. Each panel shows samples, from top to bottom, after 1 day (replicates 1 and 2), after 2 days (replicates 1 and 2), after 3 days (replicates 1 and 2), after 8 days (replicates 1 and 2) and after 10 days (replicates 1 and 2).

Figure 3 shows the MALDI-TOF MS spectra obtained from *P. digitatum* IMI 380881 grown for a shorter time period and with sampling at higher frequency, with growth for 1 day, 2 days, 3 days, 8 days and 10 days at 23°C in 10 µl droplets of CZ medium overlaid with 30 µl of light mineral oil in 1.5 ml Eppendorf tubes, on CZ agar plates, and on PDA plates.

As can be seen in Fig. 3, for the samples grown in 5 µl droplets of CZ medium overlaid with 20 µl of light mineral oil in 1.5 ml Eppendorf tubes (Fig. 3a), the spectra also change over the shorter time course, with peaks of around 8000 Da and 4000 Da that are only visible at 2 days and 3 days and a peak at around 2200 Da that is more abundant at 8 days and 10 days. For the samples grown on CZ agar plates (Fig. 3b), the spectra also change over the shorter time course, with a cluster of peaks with mass between 2000 Da and 2800 Da that are more abundant at 1 day. For the samples grown on PDA plates (Fig. 3c), the spectral consistency over the shorter time course is again better than for the samples grown in 5 µl droplets of CZ medium overlaid with 20 µl of light mineral oil in 1.5 ml Eppendorf tubes and the samples grown on CZ agar plates, with the main variance being the cluster of peaks with mass between 7500 Da and 10,000 Da, which are more abundant at 8 days and 10 days. As observed above, the number of peaks for the samples grown on PDA plates is greater than for the samples grown on CZ plates, which is in turn greater than for the samples grown in 5 µl droplets of CZ medium overlaid with 20 µl of light mineral oil in 1.5 ml Eppendorf tubes. Duplicate negative controls were carried out for all time points and all culture conditions, and again gave no spectra (data not shown).

**Figure 4:**
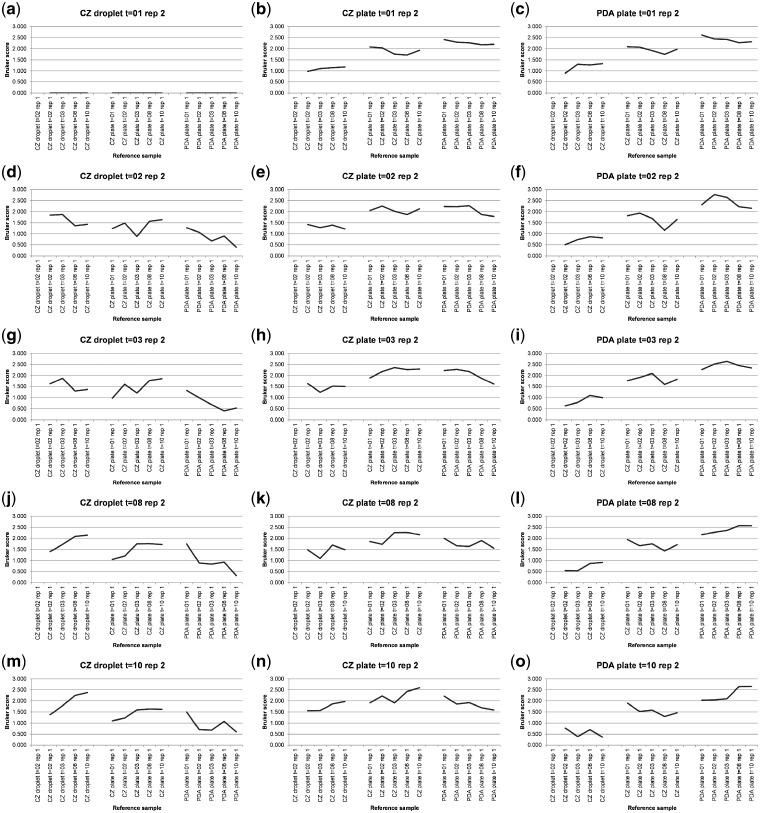
Bruker scores from comparisons between *P. digitatum* IMI 380881 replicate 1 ‘reference’ database spectra (*x*-axis labels) and replicate 2 ‘test’ sample spectra (graph titles).

Supplementary Table S2 shows MALDI-TOF MS spectral-comparison scores from the spectra shown in Fig. 3 between replicate 1 ‘reference’ database spectra and replicate 2 ‘test’ sample spectra for *P. digitatum* IMI 380881 and Fig. 4 shows Bruker scores using the data from Supplementary Table S2 for comparisons between replicate 1 ‘reference’ database spectra and replicate 2 ‘test’ sample spectra for *P. digitatum* IMI 380881.

As can be seen in Fig. 4, no reference-sample spectra or test-sample spectra were obtained for CZ liquid cultures after 1 day (Fig. 4a). For the remaining CZ liquid-culture samples, the highest Bruker score is observed for the same growth medium, growth conditions and growth time in two comparisons (Fig. 4g and Fig. 4m) and the second-highest Bruker score is observed for the same growth medium, growth conditions and growth time in two comparisons (Fig. 4d and Fig. 4j). For the CZ agar plate samples and the PDA plate samples, the highest Bruker score is in every comparison observed for the same growth medium, growth conditions, and growth time. For the nine CZ agar-plate and CZ liquid-droplet test-sample spectral comparisons against PDA plate reference samples excluding Fig. 4a, in eight cases (with the exception of Fig. 4h), the highest Bruker score is to the PDA plate grown for the shortest amount of time, again suggesting that spectral divergence from CZ medium for samples grown of PDA plates increases with growth time. In Fig. 4h, the second-highest Bruker score is to the PDA plate grown for the shortest amount of time.

Looking at the 10-day time course #2 data in the context of the Bruker scoring system, for the PDA-plates dataset, the highest Bruker score is 2.771 for the 2-day test spectrum against the 2-day reference spectrum and the lowest Bruker score is 2.026 for the 10-day test spectrum against the 1-day reference spectrum (a difference of 0.745 Bruker units, from ‘highly-probable species-level identification’ to ‘secure genus-level identification and probable species-level identification’). For the CZ-plate dataset, the highest Bruker score is 2.587 for the 10-day test spectrum against the 10-day reference spectrum and the lowest Bruker score is 1.716 for the 1-day test spectrum against the 8-day reference spectrum (a difference of 0.871 Bruker units, from ‘highly-probable species-level identification’ to ‘probable genus-level identification’). For the CZ-droplet dataset, the highest Bruker score is 2.378 for the 10-day test spectrum against the 10-day reference spectrum and the lowest Bruker score (for which spectra were obtained) is 1.301 for the 3-day test spectrum against the 8-day reference spectrum (a difference of 1.077 Bruker units, from ‘highly-probable species-level identification’ to ‘no reliable identification’). Finally, for the entire dataset, the highest Bruker score is 2.771 for the 2-day PDA-plate test spectrum against the 2-day PDA-plate reference spectrum and the lowest Bruker score is 0.322 for the 8-day CZ-droplet test spectrum against the 10-day PDA-plate reference spectrum (a difference of 2.449 Bruker units, from ‘highly-probable species-level identification’ to ‘no reliable identification’).

### Time course #3

**Figure 5:**
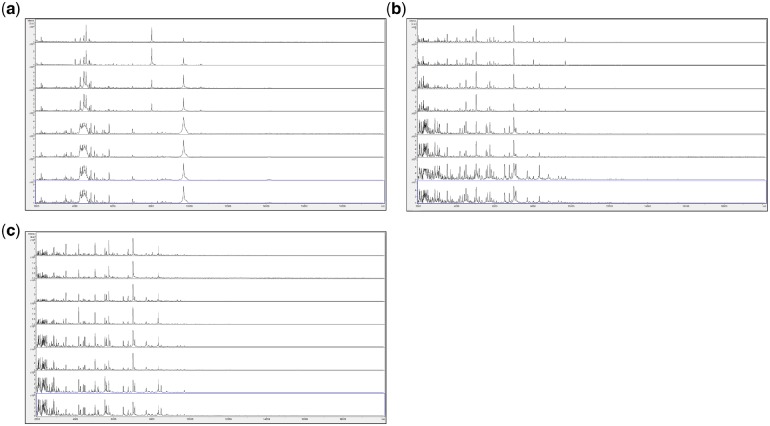
MALDI-TOF MS spectra obtained from *P. digitatum* IMI 380881 grown for 2 days, 3 days, 7 days and 10 days at 23°C (a) in 10 µl droplets of SDM (without oil overlay) in 1.5 ml Eppendorf tubes, (b) on SDM agar plates and (c) on PDA plates. Each panel shows samples, from top to bottom, after 2 days (replicates 1 and 2), after 3 days (replicates 1 and 2), after 7 days (replicates 1 and 2), and after 10 days (replicates 1 and 2).

Guided by the above observations, we next sought to formulate a more nutrient-rich liquid-culture medium that would be compatible with MALDI-TOF MS analysis. As a minimal basis, we opted to retain the components of CZ medium as these are clearly sufficient for fungal growth. As additional amino acid and vitamin sources, we empirically tested a number of standard microbiology culture-medium ingredients for the appearance of spurious MALDI-TOF MS peaks (data not shown). Bacteriological peptone and yeast extract up to 10 g/l do not contaminate MALDI-TOF MS spectra but mycological peptone above 1.25 g/l gives rise to numerous background peaks at low molecular weight. We therefore opted to include both yeast extract and bacteriological peptone at 5 g/l each as amino acid and vitamin sources. As an alternative carbohydrate source to the 30 g/l sucrose in CZ medium, we also opted to included glucose at 10 g/l. In the studies above, we also encountered a certain amount of inconvenience with the transfer of samples from under mineral-oil overlays as small volumes of oil contaminating pipette tips can occasionally leave a thin film of oil on the matrix crystals prior to analysis in the mass spectrometer. At normal fungal-growth temperatures, the water content of air at 100% humidity is around 25 g/m^3^ [22]. This means that the internal volume of a capped 1.5 ml Eppendorf tube can only retain around 38 nl of water at 100% humidity, which is an insignificant volume loss from even a 10 µl incubation volume. The real problem to solve is therefore not volume loss due to evaporation into the 1.5 ml space within the Eppendorf tube but re-condensation to form droplets within the tube any place (e.g. on the side walls) other than at the bottom where we are trying to grow fungus, a problem that can readily be solved by avoiding any temperature gradients across the tube during incubation. Informed by the above, we therefore opted to avoid mineral-oil overlays for further studies. Figure 5 shows the MALDI-TOF MS spectra obtained from *P. digitatum* IMI 380881 grown for 2 days, 3 days, 7 days and 10 days at 23°C in 10 µl droplets of the above much more nutrient-rich medium, which we have termed ‘supplements-and-dextrose medium’ (SDM), used now without an oil overlay in 1.5 ml Eppendorf tubes, on SDM agar plates, and on PDA plates.

As can be seen in Figure 5, for the samples grown in 10 µl droplets of SDM (without oil overlay) in 1.5 ml Eppendorf tubes (Fig. 5a), the spectra change over the time course, with a peak of around 8000 Da that is only visible at 2 days and 3 days and a cluster of peaks with mass between 5000 and 6000 Da that is more abundant at 7 days and 10 days. In addition, the samples grown in 10 µl droplets of SDM were observed to be very viscous and difficult to mix with the matrix-containing lysis solution. For the samples grown on SDM agar plates (Fig. 5b), the spectra change over the time course, with a visible increase in peak richness and intensity at 7 days and a still further increase in peak richness and intensity at 10 days. For the samples grown on PDA plates (Fig. 5c), the spectral consistency over the time course is again better than the other samples, with the main variance being a small increase in peak richness and intensity at 7 days and a further small increase in peak richness and intensity at 10 days. Once again, the number of peaks observed for the samples grown on agar plates is greater than the number for the samples grown in liquid culture. Duplicate negative controls were carried out for all time points and all culture conditions, and again gave no spectra (data not shown).

**Figure 6:**
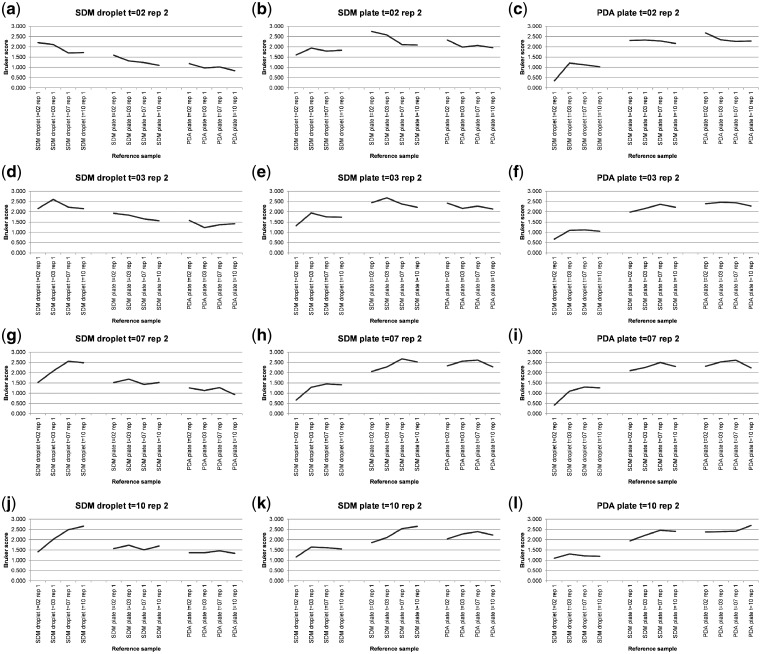
Bruker scores from comparisons between *P. digitatum* IMI 380881 replicate 1 ‘reference’ database spectra (*x*-axis labels) and replicate 2 ‘test’ sample spectra (graph titles).

Supplementary Table S3 shows MALDI-TOF MS spectral-comparison scores from the spectra shown in Fig. 5 between replicate 1 ‘reference’ database spectra and replicate 2 ‘test’ sample spectra for *P. digitatum* IMI 380881 and Fig. 6 shows Bruker scores using the data from Supplementary Table S3 for comparisons between replicate 1 ‘reference’ database spectra and replicate 2 ‘test’ sample spectra for *P. digitatum* IMI 380881.

As can be seen in Fig. 6, for the SDM liquid-droplet samples, the SDM agar plate samples, and the PDA plate samples, the highest Bruker score is in every comparison observed for the same growth medium, growth conditions and growth time. In only half of the SDM agar-plate and SDM liquid-droplet test-sample spectral comparisons against PDA plate reference samples (Fig. 6a–b and Fig. 6d–e), the highest Bruker score is to the PDA plate grown for the shortest amount of time, suggesting that spectral divergence from SDM medium for samples grown of PDA plates increases with growth time less markedly compared to CZ medium.

Looking at the 10-day time course #3 data in the context of the Bruker scoring system, for the PDA-plates dataset, the highest Bruker score is 2.690 for the 10-day test spectrum against the 10-day reference spectrum and the lowest Bruker score is 2.233 for the 7-day test spectrum against the 10-day reference spectrum (a difference of 0.457 Bruker units, from ‘highly-probable species-level identification’ to ‘secure genus-level identification and probable species-level identification’). For the SDM-plate dataset, the highest Bruker score is 2.749 for the 2-day test spectrum against the 2-day reference spectrum and the lowest Bruker score is 1.860 for the 10-day test spectrum against the 2-day reference spectrum (a difference of 0.889 Bruker units, from ‘highly-probable species-level identification’ to ‘probable genus-level identification’). For the SDM-droplet dataset, the highest Bruker score is 2.656 for the 10-day test spectrum against the 10-day reference spectrum and the lowest Bruker score is 1.412 for the 10-day test spectrum against the 2-day reference spectrum (a difference of 1.244 Bruker units, from ‘highly-probable species-level identification’ to ‘no reliable identification’). Finally, for the entire dataset, the highest Bruker score is 2.749 for the 2-day SDM-plate test spectrum against the 2-day SDM-plate reference spectrum and the lowest Bruker score is 0.344 for the 2-day PDA-plate test spectrum against the 2-day SDM-droplet reference spectrum (a difference of 2.405 Bruker units, from ‘highly-probable species-level identification’ to ‘no reliable identification’).

### Time course #4

**Figure 7:**
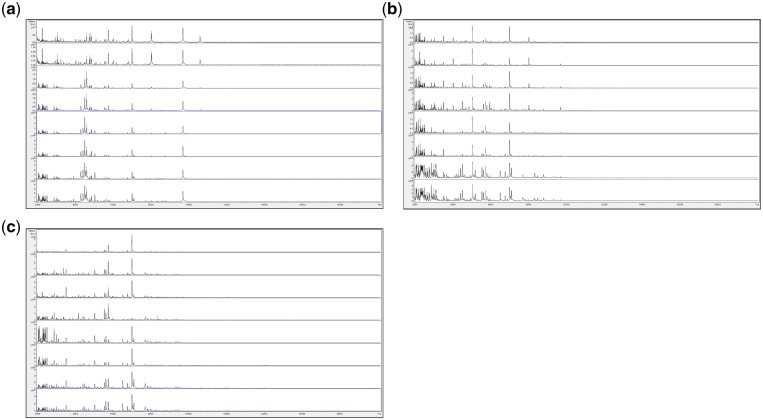
MALDI-TOF MS spectra obtained from *P. digitatum* IMI 380881 grown for 2 days, 3 days, 7 days and 10 days at 23°C (a) in 10 µl droplets of SDM absorbed into 6 mm filter-paper discs in 1.5 ml Eppendorf tubes, (b) on SDM agar plates and (c) on PDA plates. Each panel shows samples, from top to bottom, after 2 days (replicates 1 and 2), after 3 days (replicates 1 and 2), after 7 days (replicates 1 and 2) and after 10 days (replicates 1 and 2).

Guided by the above, we finally opted for a method in which fungi are grown in a rich medium which does not generate additional MALDI-TOF MS peaks, in which the entire grown fungal biomass is extracted for MALDI-TOF MS analysis, in which growth by spreading from a central point of inoculation is avoided, and in which growth in a small aqueous volume is supported on a solid matrix (6 mm filter paper discs, cut out using a hole punch) within sealed 1.5 ml Eppendorf tubes—conditions which may also favour higher rates of oxygen transfer to growing biomass than previous growth in liquid culture. Figure 7 shows the MALDI-TOF MS spectra obtained from *P. digitatum* IMI 380881 grown for 2 days, 3 days, 7 days and 10 days at 23°C in 10 µl droplets of SDM absorbed into 6 mm filter-paper discs (see ‘Discussion’ section) in 1.5 ml Eppendorf tubes, on SDM agar plates, and on PDA plates.

Figure 7 shows that, for the samples grown in 10 µl droplets of absorbed into 6 mm filter-paper discs in 1.5 ml Eppendorf tubes (Fig. 5a), spectral consistency between duplicate samples is high but there are significant spectral changes between 2 days and 3 days. From 3 days to 10 days, however (over which time period sporulation was visible on the paper discs), spectra remain very consistent. For the samples grown on SDM agar plates (Fig. 5b), spectral consistency between duplicate samples is again high but spectral changes occur throughout the time course. For the samples grown on PDA plates (Fig. 5c), spectral consistency between duplicate samples is slightly reduced (particularly at 3 days) but spectral change throughout the time course is less than that for SDM plates. The number of peaks observed for the samples grown on agar plates is comparable the number for the samples grown of paper discs. Duplicate negative controls were carried out for all time points and all culture conditions, and again gave no spectra (data not shown).

**Figure 8:**
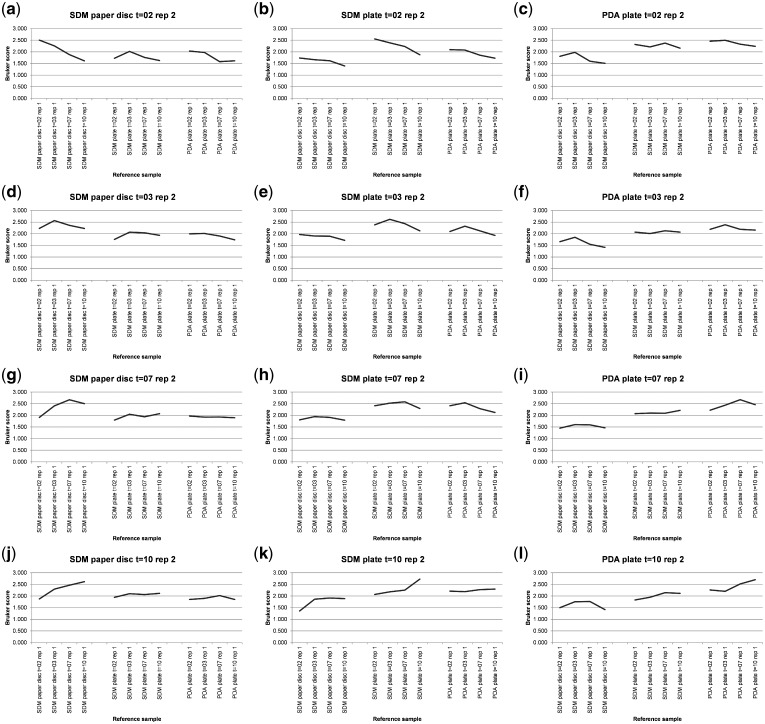
Bruker scores from comparisons between *P. digitatum* IMI 380881 replicate 1 ‘reference’ database spectra (*x*-axis labels) and replicate 2 ‘test’ sample spectra (graph titles).

Supplementary Table S4 shows MALDI-TOF MS spectral-comparison scores from the spectra shown in Fig. 7 between replicate 1 ‘reference’ database spectra and replicate 2 ‘test’ sample spectra for *P. digitatum* IMI 380881 and Fig. 8 shows Bruker scores using the data from Supplementary Table S4 for comparisons between replicate 1 ‘reference’ database spectra and replicate 2 ‘test’ sample spectra for *P. digitatum* IMI 380881.

As can be seen in Fig. 8, for the SDM liquid-droplet samples absorbed into 6 mm filter-paper discs, the SDM agar plate samples, and the PDA plate samples, the highest Bruker score is in eight out of nine comparisons observed for the same growth medium, growth conditions and growth time. The second-highest Bruker score is observed for the same growth medium, growth conditions and growth time in one comparison (Fig. 8c). In only three of the eight SDM agar-plate and SDM liquid-droplet samples absorbed into 6 mm filter-paper discs spectral comparisons against PDA plate reference samples (Fig. 8a, b and g), the highest Bruker score is to the PDA plate grown for the shortest amount of time, suggesting that spectral divergence from SDM medium for samples grown of PDA plates increases with growth time even less markedly than in the other three time courses.

Looking at the 10-day time course #4 data in the context of the Bruker scoring system, for the PDA-plates dataset, the highest Bruker score is 2.700 for the 10-day test spectrum against the 10-day reference spectrum and the lowest Bruker score is 1.578 for the 3-day test spectrum against the 10-day reference spectrum (a difference of 1.122 Bruker units, from ‘highly-probable species-level identification’ to ‘no reliable identification’). For the SDM-plate dataset, the highest Bruker score is 2.727 for the 10-day test spectrum against the 10-day reference spectrum and the lowest Bruker score is 1.875 for the 2-day test spectrum against the 10-day reference spectrum (a difference of 0.852 Bruker units, from ‘highly-probable species-level identification’ to ‘probable genus-level identification’). For the SDM paper disc dataset, the highest Bruker score is 2.666 for the 7-day test spectrum against the 7-day reference spectrum and the lowest Bruker score is 1.612 for the 2-day test spectrum against the 10-day reference spectrum (a difference of 1.054 Bruker units, from ‘highly-probable species-level identification’ to ‘no reliable identification’). Finally, for the entire dataset, the highest Bruker score is 2.727 for the 10-day SDM plate test spectrum against the 10-day SDM plate reference spectrum and the lowest Bruker score is 1.356 for the 10-day SDM plate test spectrum against the 2-day SDM paper disc reference spectrum (a difference of 1.371 Bruker units, from ‘highly-probable species-level identification’ to ‘no reliable identification’).

The highest average Bruker scores and the lowest CVs (standard deviations divided by means, expressed as a percentage) for Bruker scores from all spectral comparisons are shown in Table 1.

**Table 1:** Highest average Bruker scores and the lowest CVs (standard deviations divided by means, expressed as a percentage) for Bruker scores from all spectral comparisons. Averages were calculated from the datasets in the tables (Table S2–Table S4) specified in the first column and the growth conditions (CZ droplet, SDM droplet, SDM paper disc, SDM plate and PDA plate) also specified in the first column, over 2–10 days and also over 3–10 days.

Experimental samples (over 2–10 days)	Average Bruker score from all spectral comparisons	CV for Bruker scores from all spectral comparisons (%)
CZ droplet (from Table S2 data)	1.738	20.024
SDM droplet (from Table S3 data)	2.131	17.974
SDM paper disc (from Table S4 data)	2.274	13.482
Agar-plate controls (over 2-10 days)		
SDM plate (from Table S3 and Table S4 data)	2.362	10.443
PDA plate (from Table S2, Table S3, and Table S4 data)	2.407	7.571
Experimental samples (over 3–10 days)		
CZ droplet (from Table S2 data)	1.878	19.943
SDM droplet (from Table S3 data)	2.363	10.300
SDM paper disc (from Table S4 data)	2.456	5.958
Agar-plate controls (over 3–10 days)		
SDM plate (from Table S3 and Table S4 data)	2.433	8.513
PDA plate (from Table S2, Table S3 and Table S4 data)	2.448	7.148

For all experimental growth conditions and agar-plate controls combined, the highest average Bruker score from all spectral comparisons is 2.456 for SDM paper discs over 3–10 days and the lowest CV for Bruker scores from all spectral comparisons is 5.958%, also for SDM paper discs over 3–10 days. The lowest average Bruker score from all spectral comparisons is 1.738 for CZ droplets over 2–10 days and the highest CV for Bruker scores from all spectral comparisons is 20.024%, also for CZ droplets over 2–10 days. For just the agar-plate controls, the highest average Bruker score from all spectral comparisons is 2.448 for PDA plates over 3–10 days and the lowest CV for Bruker scores from all spectral comparisons is 7.148%, also for PDA plates over 3–10 days. The lowest agar-plate-control average Bruker score from all spectral comparisons is 2.362 for SDM plates over 2–10 days and the highest CV for Bruker scores from all spectral comparisons is 10.443%, also for SDM plates over 2–10 days. It must however be noted that, while the highest average Bruker score from all spectral comparisons is 2.448 for PDA plates over 3–10 days using the combined data from Supplementary Tables S2–S4, higher average Bruker scores were found within some of the un-combined datasets (2.482 for PDA plates over 3–10 days using just the data from Table S2 and 2.450 for SDM plates over 3–10 days using just the data from Supplementary Table S3, the first of which is higher than the average Bruker score of 2.456 for SDM paper discs grown over 3–10 days). Likewise, whilst the lowest CV for Bruker scores from all spectral comparisons is 7.148 for PDA plates over 3–10 days using the combined data from Supplementary Table S2–S4, lower CVs were found within some of the uncombined datasets (5.916% for PDA plates over 2–10 days using just the data from Supplementary Table S3 and 5.930% for PDA plates over 2–10 days using just the data from Table S3, both of which are lower than the CV of 5.958% for SDM paper discs grown over 3–10 days).

## Discussion and conclusions

Through the analysis of highly-expressed acid-soluble sub-proteomes, MALDI-TOF MS is a powerful and versatile tool for the characterization and identification of protein-containing samples, including filamentous fungi, which are the focus of the current study. For the MALDI-TOF MS analysis of fungi, it is desirable to have not only a means of protein extraction and MALDI-TOF MS sample preparation that is reliable, cheap, and simple to perform but also a means of fungal growth in which the resulting MALDI-TOF MS spectrum remains as consistent as possible, despite real-life variations in fungal growth rates, adaptation, and/or incubation times. For the former, we have employed a previously developed, highly simplified and inexpensive method (with broad applicability also to bacteria, insects, and plants [12, 13]) that lyses cells by immersion in aqueous acetonitrile containing TFA to selectively extract acid-soluble proteins, with lysis and extraction carried out in the presence of near-saturated and inexpensive-grade MALDI matrix. The resulting matrix-saturated lysate containing acid-solubilized proteins is then simply dried down directly onto the MALDI-TOF MS sample plate and analysed. For the latter, we initially grew fungi for up to 3 weeks in very small volumes of CZ medium containing antibiotics, under an oil overlay within capped Eppendorf tubes, with subsequent use of the entire liquid volume for MALDI-TOF MS sample preparation. In spite of our initial thought process, using this method we observed marked spectral changes over growth time, along with lower peak richness compared to PDA-plate controls. Similar results were obtained for the same culturing conditions over a shorter time course, with growth for up to 10 days and more frequent sampling. We next opted for growth in small-volume liquid culture using a more nutrient-rich medium that is compatible with MALDI-TOF MS, now without an oil overlay but, using this revised method, we again observed marked spectral changes for the samples grown in liquid culture. For the control samples grown on PDA plates, the spectral consistency over the time course was once again better than the other samples, and once again, the number of peaks observed for the samples grown on agar plates was greater than the number for the samples grown in liquid culture. Guided by the above, we finally opted for growth in a small aqueous volume supported on a solid matrix (6 mm filter paper discs, cut out using a hole punch) within sealed 1.5 ml Eppendorf. Using this final method, whilst we observed significant spectral changes between 2 days and 3 days, from 3 days to 10 days the spectra remained very consistent, with the number of peaks observed for the samples grown on agar plates comparable to the number for the samples grown of paper discs. Spectral divergence from samples grown on PDA plates also increased with growth time less markedly than in the other three time courses.

For all experimental growth conditions and agar-plate controls combined, the highest average Bruker score from all spectral comparisons was for SDM paper discs over 3–10 days and the lowest CV for Bruker scores from all spectral comparisons was also for SDM paper discs over 3–10 days. While this is true using the combined data from Supplementary Tables S2–S4, higher average Bruker scores and lower CVs were found within some of the uncombined datasets for the agar-plate controls, allowing only a tentative conclusion that the SDM paper-disc method is slightly better than PDA-plate controls. With this in mind, we speculate that a better solution might perhaps be to accept that spectral variation during fungal growth is difficult to eliminate and instead to minimize this in a manner that facilitates the construction of growth-time-point specific databases for fungal identification. Current practice requires standardized protocols for reliable identifications, along with databases constructed using exactly the same method as for the identification of unknown samples. While such databases are not currently available for any of the above methods, the SDM paper-disc method might be well suited to the *de novo* construction of such databases because it minimizes spectral variation as well as if not slightly better than PDA plates and is a considerably easier format than agar plates for preparing spectra from fungal strains at multiple time points for growth-time-point specific database construction, which is an approach that we plan to investigate in future studies. In the above study, we have only employed a single reference and a single comparison sample for each time point. For the construction of a growth-time-point specific database, we would also advocate the use of multiple inocula and multiple extractions per time point in order to accommodate better these main sources of variance.

The current study is limited to a single species (*P. digitatum*), with growth of controls on PDA plates. Future studies will aim to investigate whether the results obtained with the above species are generally applicable, whether SDM is suitable for growth of other fungal species (and also reduces spectral variability when grown in paper-supported culture), along with comparison to controls carried out on media other than PDA (e.g. Sabouraud agar and broth, which are frequently employed for fungal growth prior to MALDI-TOF MS analysis [3, 10, 11]).

## Data availability

Original spectral data held on the Bruker Microflex PC is available on request.

## Supplementary Material

Supplementary Data
