# Supplementary material for: A method for filamentous fungal growth and sample preparation aimed at more consistent MALDI-TOF MS spectra despite variations in growth rates and/or incubation times
Source: Biol Methods Protoc. 2019 May 3;4(1):bpz003. doi: 10.1093/biomethods/bpz003 (PMC7200990; doi:10.1093/biomethods/bpz003)
Supplement: Supplementary Data [file bpz003_supp.pdf]

Supplementary materials

Figure S1

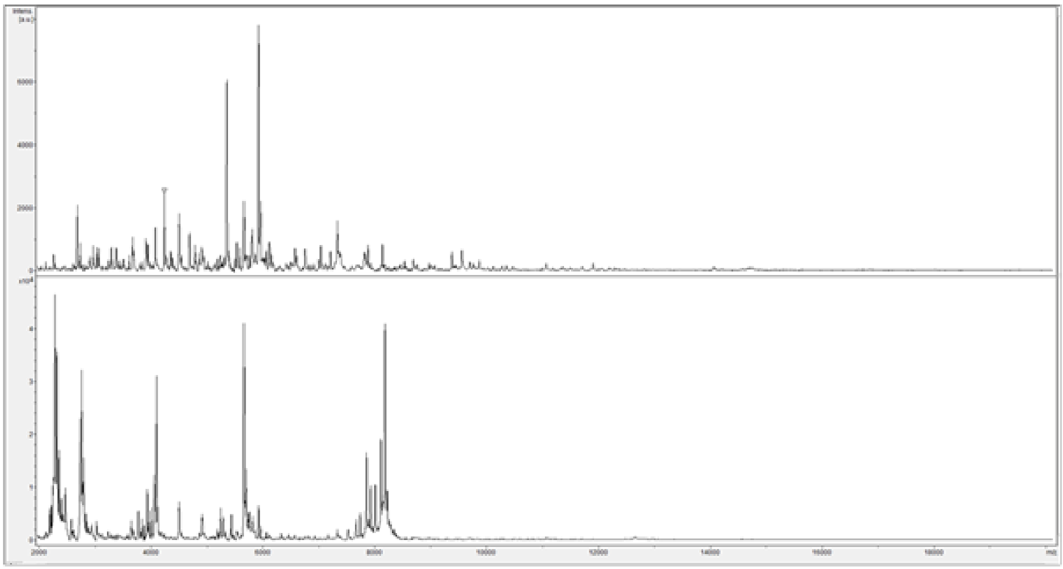

Figure S1 MALDI-TOF MS spectra from the same strain (CAV 789 [14]) of *Fusarium oxysporum* f.sp *cubense* grown for one week at 25°C on 15 g/l agar plates containing PDA Oxoid medium (4 g/l potato extract, 20 g/l glucose, pH 5.6) (top) and SNA medium (1 g/l potassium phosphate, 1 g/l potassium nitrate, 0.5 g/l magnesium sulphate, 0.5 g/l potassium chloride, 0.2 g/l glucose, 0.2 g/l sucrose, pH 6.5) (bottom).

Figure S2

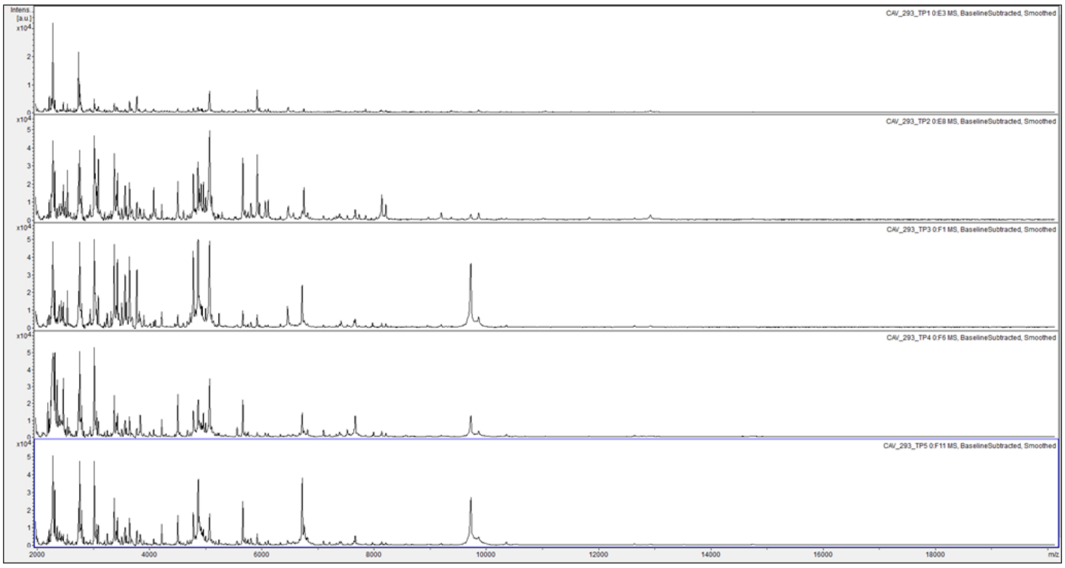

Figure S2 MALDI-TOF MS spectra from the same strain (CAV 293 [15]) of *Fusarium oxysporum* f.sp *cubense* grown at 25°C on 15g/l agar plates containing SNA medium (1 g/l potassium phosphate, 1 g/l potassium nitrate, 0.5 g/l magnesium sulphate, 0.5 g/l potassium chloride, 0.2 g/l glucose, 0.2 g/l sucrose, pH 6.5) for, from top to bottom, 3 days, 6 days, 11 days, 16 days, and 19 days.

Table S1

	CZ droplet t=01 rep 2	CZ plate t=01 rep 2	PDA plate t=01 rep 2	CZ droplet t=02 rep 2	CZ plate t=02 rep 2	PDA plate t=02 rep 2	CZ droplet t=03 rep 2	CZ plate t=03 rep 2	PDA plate t=03 rep 2
CZ droplet t=01 rep 1	2.144	1.700	1.634	1.692	1.444	0.956	1.245	1.556	0.586
CZ droplet t=02 rep 1	1.539	1.418	1.460	2.371	1.238	0.816	1.892	1.577	0.698
CZ droplet t=03 rep 1	1.715	1.733	1.587	2.019	1.528	0.899	2.076	1.781	0.611
CZ plate t=01 rep 1	1.544	2.507	2.311	1.726	2.500	2.137	1.417	2.290	1.795
CZ plate t=02 rep 1	1.433	2.408	2.158	1.983	2.410	2.078	1.617	2.510	1.902
CZ plate t=03 rep 1	1.361	2.152	2.071	1.742	1.976	1.794	1.681	2.632	1.654
PDA plate t=01 rep 1	1.304	2.134	2.767	1.943	2.222	2.335	1.632	2.143	2.180
PDA plate t=02 rep 1	0.793	1.870	2.389	1.363	2.073	2.746	1.090	1.882	2.524
PDA plate t=03 rep 1	0.611	1.604	1.896	1.142	1.766	2.295	0.968	1.794	2.350

Table S1 *P. digitatum* IMI 380881 MALDI-TOF MS spectral-comparison scores between replicate 1 ‘reference’ database spectra (column 1) and replicate 2 ‘test’ sample spectra (row 1).

Table S2

	CZ droplet t=01 rep 2	CZ plate t=01 rep 2	PDA plate t=01 rep 2	CZ droplet t=02 rep 2	CZ plate t=02 rep 2	PDA plate t=02 rep 2	CZ droplet t=03 rep 2	CZ plate t=03 rep 2	PDA plate t=03 rep 2	CZ droplet t=08 rep 2	CZ plate t=08 rep 2	PDA plate t=08 rep 2	CZ droplet t=10 rep 2	CZ plate t=10 rep 2	PDA plate t=10 rep 2
CZ droplet t=01 rep 1	0.000	0.000	0.000	0.000	0.000	0.000	0.000	0.000	0.000	0.000	0.000	0.000	0.000	0.000	0.000
CZ droplet t=02 rep 1	0.000	0.979	0.900	1.842	1.425	0.511	1.628	1.630	0.632	1.401	1.476	0.545	1.374	1.557	0.769
CZ droplet t=03 rep 1	0.000	1.105	1.300	1.872	1.279	0.735	1.879	1.244	0.783	1.721	1.090	0.535	1.780	1.563	0.389
CZ droplet t=08 rep 1	0.000	1.147	1.272	1.366	1.398	0.865	1.301	1.512	1.103	2.085	1.692	0.868	2.241	1.860	0.703
CZ droplet t=10 rep 1	0.000	1.179	1.333	1.430	1.215	0.817	1.369	1.504	0.997	2.144	1.487	0.915	2.378	1.970	0.363
CZ plate t=01 rep 1	0.000	2.081	2.093	1.235	2.053	1.819	0.977	1.897	1.771	1.045	1.856	1.939	1.101	1.910	1.895
CZ plate t=02 rep 1	0.000	2.043	2.073	1.491	2.257	1.936	1.602	2.179	1.915	1.197	1.726	1.663	1.224	2.219	1.519
CZ plate t=03 rep 1	0.000	1.754	1.917	0.883	2.021	1.700	1.213	2.361	2.097	1.747	2.261	1.748	1.594	1.909	1.581
CZ plate t=08 rep 1	0.000	1.716	1.745	1.571	1.868	1.154	1.772	2.268	1.594	1.753	2.266	1.432	1.631	2.422	1.294
CZ plate t=10 rep 1	0.000	1.931	1.981	1.646	2.130	1.660	1.857	2.295	1.835	1.714	2.165	1.709	1.620	2.587	1.461
PDA plate t=01 rep 1	0.000	2.412	2.618	1.275	2.237	2.319	1.328	2.228	2.268	1.742	1.997	2.164	1.496	2.212	2.026
PDA plate t=02 rep 1	0.000	2.292	2.439	1.068	2.231	2.771	0.993	2.280	2.524	0.890	1.660	2.273	0.704	1.855	2.042
PDA plate t=03 rep 1	0.000	2.266	2.420	0.674	2.272	2.645	0.681	2.179	2.647	0.841	1.633	2.358	0.682	1.918	2.093
PDA plate t=08 rep 1	0.000	2.181	2.268	0.896	1.874	2.227	0.404	1.878	2.453	0.929	1.898	2.575	1.074	1.690	2.647
PDA plate t=10 rep 1	0.000	2.201	2.310	0.397	1.789	2.158	0.533	1.616	2.344	0.322	1.555	2.571	0.604	1.589	2.648

Table S2 *P. digitatum* IMI 380881 MALDI-TOF MS spectral-comparison scores between replicate 1 ‘reference’ database spectra (column 1) and replicate 2 ‘test’ sample spectra (row 1).

Table S3

	SDM droplet t=02 rep 2	SDM plate t=02 rep 2	PDA plate t=02 rep 2	SDM droplet t=03 rep 2	SDM plate t=03 rep 2	PDA plate t=03 rep 2	SDM droplet t=07 rep 2	SDM plate t=07 rep 2	PDA plate t=07 rep 2	SDM droplet t=10 rep 2	SDM plate t=10 rep 2	PDA plate t=10 rep 2
SDM droplet t=02 rep 1	2.199	1.602	0.344	2.157	1.318	0.670	1.526	0.662	0.420	1.412	1.158	1.093
SDM droplet t=03 rep 1	2.113	1.936	1.208	2.604	1.941	1.093	2.078	1.284	1.099	2.018	1.644	1.308
SDM droplet t=07 rep 1	1.702	1.792	1.122	2.224	1.752	1.120	2.555	1.452	1.297	2.489	1.614	1.212
SDM droplet t=10 rep 1	1.718	1.829	1.025	2.156	1.734	1.051	2.484	1.411	1.258	2.656	1.551	1.191
SDM plate t=02 rep 1	1.591	2.749	2.307	1.925	2.444	1.980	1.514	2.058	2.096	1.568	1.860	1.943
SDM plate t=03 rep 1	1.310	2.575	2.331	1.840	2.680	2.162	1.685	2.281	2.252	1.730	2.106	2.216
SDM plate t=07 rep 1	1.232	2.110	2.285	1.650	2.374	2.363	1.428	2.676	2.497	1.507	2.543	2.459
SDM plate t=10 rep 1	1.101	2.088	2.162	1.566	2.220	2.220	1.518	2.522	2.300	1.693	2.651	2.405
PDA plate t=02 rep 1	1.181	2.329	2.674	1.581	2.420	2.390	1.251	2.334	2.312	1.367	2.038	2.376
PDA plate t=03 rep 1	0.965	1.984	2.342	1.231	2.168	2.462	1.134	2.557	2.520	1.363	2.281	2.387
PDA plate t=07 rep 1	1.015	2.067	2.258	1.363	2.277	2.443	1.270	2.613	2.607	1.457	2.391	2.413
PDA plate t=10 rep 1	0.836	1.954	2.280	1.420	2.137	2.280	0.930	2.285	2.233	1.337	2.231	2.690

Table S3 *P. digitatum* IMI 380881 MALDI-TOF MS spectral-comparison scores between replicate 1 ‘reference’ database spectra (column 1) and replicate 2 ‘test’ sample spectra (row 1).

Table S4

	SDM paper disc t=02 rep 2	SDM plate t=02 rep 2	PDA plate t=02 rep 2	SDM paper disc t=03 rep 2	SDM plate t=03 rep 2	PDA plate t=03 rep 2	SDM paper disc t=07 rep 2	SDM plate t=07 rep 2	PDA plate t=07 rep 2	SDM paper disc t=10 rep 2	SDM plate t=10 rep 2	PDA plate t=10 rep 2
SDM paper disc t=02 rep 1	2.505	1.736	1.810	2.230	1.969	1.659	1.912	1.804	1.448	1.874	1.356	1.501
SDM paper disc t=03 rep 1	2.258	1.662	1.981	2.566	1.903	1.850	2.407	1.943	1.602	2.297	1.865	1.753
SDM paper disc t=07 rep 1	1.881	1.618	1.599	2.363	1.897	1.545	2.666	1.910	1.589	2.463	1.916	1.766
SDM paper disc t=10 rep 1	1.612	1.393	1.507	2.229	1.717	1.417	2.500	1.791	1.463	2.615	1.888	1.414
SDM plate t=02 rep 1	1.726	2.554	2.319	1.758	2.382	2.070	1.793	2.408	2.069	1.942	2.066	1.832
SDM plate t=03 rep 1	2.019	2.388	2.213	2.066	2.622	2.009	2.045	2.522	2.097	2.099	2.180	1.948
SDM plate t=07 rep 1	1.762	2.232	2.375	2.041	2.436	2.128	1.935	2.579	2.090	2.062	2.253	2.142
SDM plate t=10 rep 1	1.626	1.875	2.158	1.932	2.124	2.071	2.072	2.294	2.211	2.113	2.727	2.113
PDA plate t=02 rep 1	2.034	2.095	2.457	1.999	2.098	2.192	1.967	2.411	2.221	1.854	2.210	2.259
PDA plate t=03 rep 1	1.971	2.077	2.496	2.012	2.326	2.388	1.920	2.542	2.433	1.898	2.189	2.201
PDA plate t=07 rep 1	1.578	1.855	2.330	1.905	2.124	2.192	1.925	2.284	2.671	2.021	2.274	2.517
PDA plate t=10 rep 1	1.614	1.730	2.237	1.741	1.930	2.159	1.897	2.121	2.457	1.853	2.298	2.700

Table S4 *P. digitatum* IMI 380881 MALDI-TOF MS spectral-comparison scores between replicate 1 ‘reference’ database spectra (column 1) and replicate 2 ‘test’ sample spectra (row 1).
